# The Inclusion of Pea in Concentrates Had Minor Effects on the Meat Quality of Light Lambs

**DOI:** 10.3390/ani11082385

**Published:** 2021-08-12

**Authors:** Mireia Blanco, Guillermo Ripoll, Sandra Lobón, Juan Ramón Bertolín, Isabel Casasús, Margalida Joy

**Affiliations:** Centro de Investigación y Tecnología Agroalimentaria de Aragón (CITA), Instituto Agroalimentario de Aragón–IA2 (CITA-Universidad de Zaragoza), Avda. Montañana 930, 50059 Zaragoza, Spain; gripoll@cita-aragon.es (G.R.); slobon@cita-aragon.es (S.L.); jrbertolin@cita-aragon.es (J.R.B.); icasasus@cita-aragon.es (I.C.); mjoy@cita-aragon.es (M.J.)

**Keywords:** *Pisum sativum*, fatty acids, colour, texture, soybean

## Abstract

**Simple Summary:**

The use of local protein sources, such as pea (*Pisum sativum*), has been encouraged to reduce the dependency of Europe on soybean meal imports. Changes in the ingredients of iso-energetic concentrates may affect the fatty acid profiles of the concentrates and other secondary compounds and, therefore, affect meat quality parameters. The objective of the study was to compare the carcass colour, and the meat quality parameters (texture, chemical composition, lipid oxidation, fatty acids) of lambs fed concentrates with different proportions of pea for replacing soybean meal. The inclusion of pea had no effects on carcass colour and minor effects on the fatty acid profile. Therefore, the inclusion of pea can be recommended to increase the self-sufficiency of lamb production systems.

**Abstract:**

The use of pea (*Pisum sativum*) has been recommended to replace soybean meal in the diet of ruminants, but it may affect meat quality. The aim of this study was to evaluate the effect of the proportion of pea (0%, 10%, 20% and 30%) in fattening concentrates fed to light lambs for 41 days on carcass colour and on the meat quality. Pea inclusion affected neither the colour of the lamb carcasses nor affected most of the parameters of the meat quality. However, the inclusion of pea affected the cholesterol content, and the 20%pea concentrate yielded meat with greater cholesterol contents than the 30%pea concentrate did (*p* < 0.05). The inclusion of pea had minor effects on individual FAs but affected the total saturated fatty acids (*p* < 0.01) and the thrombogenicity index (*p* < 0.05). A greater total saturated fatty acid content was recorded for the 20%pea concentrate than for the rest of the concentrates, and a greater thrombogenicity index was recorded for the 20% concentrate than for the 10%pea concentrate. The results indicated the viability of the inclusion of pea in the fattening concentrate of light lambs without impairing meat quality, with the 30%pea concentrate being the most suitable to reduce the soya-dependency.

## 1. Introduction

There is a worldwide interest in enhancing the use of pea (*Pisum sativum* L.) in the diets of ruminants. Using pea in place of soybean meal (SBM) has been encouraged to reduce dependency on imports in Europe [1]. Moreover, European consumers reject soybean, as it is mainly a genetically modified organism [2] and is therefore banned by organic production regulations [3] and because of sustainability issues linked to deforestation in soybean production areas [4].

Pea has a high crude protein (CP) content [5,6] and highly soluble and degradable starch [7], and the net energy for the weight gain of pea is at least equal to that of corn and is greater than that of SBM [6]. Up to 15% pea inclusion in the fattening diets of light lambs is recommended [8], but the use of pea has been studied scarcely. Nevertheless, minor effects were observed when lambs were fattening with greater rates of pea inclusion [9,10,11,12]. Purroy, et al. [13] observed greater internal fat depositions in lambs that were fed concentrate including pea instead of SBM, and they related these results to the differences in the net energies for the weight gain of these protein sources. Therefore, the intramuscular fat (IMF) deposition in the studied lambs might also have been altered, although this effect was not studied. The inclusion of pea can also affect the fatty acid (FA) composition of lambs [6,12,14] and improve the health properties of meat because of the deposition of n-3 polyunsaturated fatty acids (PUFAs) is increased when pea is used compared to SBM [12,15]. Moreover, pea can be a source of carotenoid [3], which affects fat colour and lipid oxidation during storage and have a minor effect on meat colour [9]. Therefore, the aim of this study was to evaluate the effects of increasing the proportion of pea in the fattening concentrate of light lambs on the fat colour and meat quality.

## 2. Materials and Methods

The experiment was conducted in the facilities of the CITA Research Centre (41°3′ N, 0°47′ W, 216 m above sea level) in Zaragoza (Spain).

### 2.1. Animal Management and Experimental Design

At weaning, 54 male Rasa Aragonesa lambs (13.4 ± 0.16 kg in BW; 31 ± 0.6 d in age) were randomly selected among 98 single raised lambs of the experimental flock. The lambs were randomly assigned to 1 of 4 groups, balanced by BW and age at weaning and by weight gain during suckling. Each group received a pelleted concentrate with a different proportion of pea (0%, 10%, 20% or 30%) and barley straw on an ad libitum basis for 41 (±1.4) days (d), at which point they reached 23.1 (±0.11) kg in BW. The inclusion of pea mainly replaced SBM, and the pea concentrates were formulated to be iso-energetic (13.3 MJ metabolic energy kg^−1^ DM) and iso-proteic (198 g CP kg^−1^ DM) [16] considering a barley straw intake of 10%. The main ingredients were barley, corn, soybean meal, wheat, pea, wheat bran, sugarcane molasses and palm oil. Further information on the ingredients and chemical composition of concentrates, the management of the lambs during the fattening period, and information on the slaughtering procedures are reported elsewhere [11]. Each week, samples of the concentrates were collected for chemical analyses.

### 2.2. Slaughtering Conditions and Sampling

After slaughter, the carcasses were chilled at 4 °C for 24 h in total darkness. The carcasses were split along the dorsal line. The *Rectus abdominis* (RA), *Longissimus thoracis et lumborum* (LTL), *Semimembranosus* (SM) and *Semitendinosus* (ST) muscles were removed. The LTL muscle from the 4th to the 6th lumbar *Vertebrae* of the left half of the carcass was sliced, freeze-dried and minced to determine its chemical composition and retinol, cholesterol and tocopherol contents. The same portion from the right half of the carcass was identically processed to analyse the fatty acid composition. The LTL muscles from the 6th to the 13th thoracic *Vertebrae* were sliced into 2.5-cm-thick samples, which were randomly placed in trays wrapped with oxygen-permeable PVC film and kept in darkness at 4 °C until the colour was measured (0, 2, 5, 7 and 9 d of air exposure). The 0-d samples were also allowed to bloom in darkness at 4 °C for 1 h before being measured. Immediately after the colour measurements were conducted, the samples were vacuum-packed and frozen (at −20 °C) until lipid oxidation analysis. The ST and SM muscles were immediately vacuum-packed and stored at −20 °C until texture determinations.

### 2.3. Determinations

The colours of the RA and LTL muscles, subcutaneous caudal fat and perirenal fat were measured using a Minolta CM-2006d spectrophotometer (Konica Minolta Holdings, Inc., Osaka, Japan) in CIEL*a*b* space [17]. The lightness (L*), redness (a*) and yellowness (b*) were recorded and used to calculate the hue angle ((*h_ab_*) $=\tan^{-1}\left(\frac{b^{*}}{a^{*}}\right)\times 57.29$, expressed in degrees), and the chroma ((C*_ab_) $=\sqrt{\left(a^{*2}+b^{*2}\right)}$). The relative contents of metmyoglobin (MMb), oxymyoglobin (MbO_2_) and deoxymyoglobin (DMb) in the meat were estimated [18]. The absolute value of the integral of the translated spectrum (SUM) was calculated in the fat deposits [19].

The intramuscular lipid oxidation of the LTL muscle was determined following the procedure reported by Ripoll, González-Calvo, Molino, Calvo and Joy [17]. The ST muscle was used to study the shear force in cooked meat using an Instron machine model 5543 (Instron Limited, Cerdanyola, Spain). The muscles were thawed in tap water until they reached an internal temperature of 16–19 °C and then cooked in a water bath at 75 °C (internal temperature of 70 °C). The temperatures were controlled with a Testo 108-2 waterproof food thermometer with a Type T thermocouple (Instrumentos Testo S.A., Cabrils, Spain). The steaks were cooled overnight at room temperature. Meat blocks of 1 × 1 × 3 cm were sheared perpendicularly to the long axis of the block using a Warner–Bratzler device with a cross-head speed of 2.5 mm s^−1^. The shear force and toughness of each block were determined. The SM muscle was used to determine the texture in raw meat using a modified compression device that avoids the transversal elongation of the sample [20]. Cores of 30 × 10 × 10 mm were cut. The stress was measured when the probe compressed the core by 20% and 80%, with a cross-head speed of 0.83 mm s^−1^.

### 2.4. Chemical Analyses

The samples were weighed before and after freeze-drying to obtain the DM content. The CP content was determined following the Dumas procedure [21] using a nitrogen analyser (Model NA 2100, CE Instruments, Thermoquest SA, Barcelona, Spain). The IMF was determined following the Ankom procedure [22] with an XT10 Ankom extractor (Ankom Technology Corporation, New York, NY, USA). The extraction of carotenoids and tocopherols in the concentrates was performed following the procedure described in Blanco, et al. [23], whereas the extraction of retinol, tocopherols and cholesterol in the meat was performed following the methodology of Bertolín, et al. [24]. In both analyses, an Acquity UPLC H-Class liquid chromatograph (Waters, Mildford, KA, USA) equipped with a silica-based bonded phase column (Acquity UPLC HSS T3, 1.8-μm × 2.1-mm × 150-mm column, Waters, Mildford, KA, USA), an absorbance detector (Acquity UPLC Photodiode Array PDA eλ Detector; Waters, Mildford, KA, USA) and a fluorescence detector (2475 Multi λ Fluorescence Detector, Waters, Mildford, KA, USA) was used. The carotenoids, retinol and cholesterol were detected by measuring the absorbance at 450, 325 and 220 nm, respectively, and the tocopherols were detected by measuring the fluorescent emissions at λexc = 295 and λemi = 330 nm.

Fatty acids were extracted and derivatised from the concentrates (extracted with heptane) [25] and from the meat samples [26]. Fatty acid methyl esters (FAMEs) were determined using a GC (Bruker 436 Scion gas, Billerica, MA, USA) equipped with a cyanopropyl capillary column (BR-2560, 100 m × 0.25 mm ID × 0.20 µm thick, Bruker, Billerica, MA, USA) with a flame ionisation detector and Compass CDS software. FA identification was performed using the GLC-532, GLC-401, GLC-643, GLC-642, GLC-463, C18:1 t11, C19:0 and C23:0 standard references (Nu-Chek-Prep Inc., Elysian MN, USA) and the relative retention times observed in the literature [26,27]. FA quantification was performed following the UNE-EN 12966-4 Official Method (2015). After individual FA determination, the sum of the saturated fatty acids (SFA), monounsaturated FAs (MUFA), polyunsaturated FAs (PUFA), PUFA n-6, PUFA n-3 and n-6:n-3 ratio were calculated. The indexes of atherogenicity (IA) and thrombogenicity (TI) were calculated by the following formulas according to the method used by Ulbricht and Southgate [28]:

atherogenicity index

(1) $$=\frac{\left(C12:0+4\times C14:0+C16:0\right)}{\left(\sum \mathrm{MUFA}+\sum n-6+\sum n-3\right)}$$

and

thrombogenicity index

(2) $$=\frac{\left(C14:0+\;C16:0+C18:0\right)}{\left(0.5\times \sum \mathrm{MUFA}+0.5\times \sum n-6+3\times \frac{\sum n-3}{\sum n-6}\right)\;}$$

### 2.5. Statistical Analyses

The data were analysed with SAS 9.4 statistical software (SAS Inst. Inc., Cary, NC, USA). The chemical composition, tocopherol content, cholesterol content and FA composition of the LTL muscle were analysed using the GLM procedure with the pea proportion as the fixed effect. The colour and lipid oxidation of the LTL muscle were analysed with a mixed model (MIXED procedure) using repeated measurements. The pea proportion, meat display time and the interaction between the two factors were included as fixed effects, and the lamb was included as a random effect. The degrees of freedom were adjusted with the Kenward–Roger correction to account for unequal observations or missing values. To model the error, different variance-covariance matrices were tested, and the matrix with the lowest Aikake and Bayesian information criteria was chosen. Multiple comparisons among treatments were performed using Tukey’s method. The least-square smeans and standard errors were obtained, and differences were considered significant when *p <* 0.05. The trends were discussed when 0.10 < *p* ≤ 0.05.

## 3. Results

### 3.1. Feedstuffs

The main FAs in the concentrates were C16:0, C18:0, C18:1 c9 and C18:2 n-6, all of which were affected by the pea proportion (*p <* 0.001; Table 1). The percentage of C16:0 was greatest in the 20%pea concentrate, intermediate in the 10%pea and 30%pea concentrates and lowest in the 0%pea concentrate (*p <* 0.001). The percentage of C18:0 was greater in the 0%pea and 30%pea concentrates than in the other two concentrates (*p <* 0.001). The percentage of C18:1 c9 was lower in the 0%pea concentrate than in the other concentrates, whereas the percentage of C18:2 n-6 was higher in the 0%pea concentrate than in the rest of the concentrates (*p <* 0.001).

Regarding the carotenoids, lutein, zeaxanthin, all-E-, 13Z- and 9Z-β-carotene were detected in low quantities in all concentrates, with differences obtained among the concentrates (*p <* 0.001; Table 1) with no clear pattern. The lutein content increased with the pea proportion, whereas the concentrate with 0%pea had the greatest zeaxanthin content, and the concentrate with 20%pea had the greatest β-carotene content (*p <* 0.001). Contents of γ-, α- and δ-tocopherols were detected in all concentrates and were affected by the inclusion of pea. The content of γ-tocopherol increased with the proportion of pea, whereas the 20%pea concentrate presented the greatest δ-tocopherol content (*p <* 0.001) and α-tocopherol content that was only greater than that of the 30%pea concentrate.

### 3.2. Carcass Colour

The colour parameters of the studied RA muscle and fat deposits were similar among the concentrates (*p >* 0.05; Table 2).

### 3.3. Meat Quality

**Figure 1:**
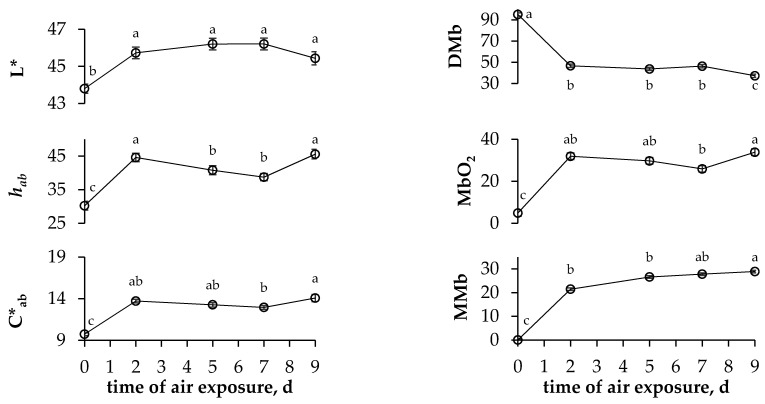
Evolution of colour parameters (lightness (L*), hue angle (***h_ab_***) and chroma (C*_ab_)) and haem pigments (deoxy-myoglobin (**DMb**), oxymyoglobin (**MbO_2_**) and metmyoglobin (**MMb**)) of LTL muscle throughout display. Vertical bars indicate the standard error of the mean; different letters indicate differences between times (*p* < 0.05).

**Figure 2:**
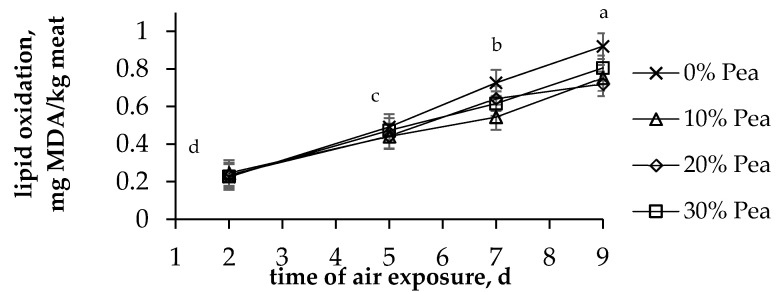
Effect of the proportion of pea in the concentrate on lipid oxidation during air exposure. Vertical bars indicate the standard error of the mean; different letters indicate differences between times (*p* < 0.001); MDA: malondialdehyde.

The colours, pigments and lipid oxidation levels of the LTL muscle were only affected by the time of air exposure (*p <* 0.001), nor by pea proportion, neither by the interaction of time and pea proportion (*p* > 0.05). All colour parameters increased between 0 and 2 d (*p <* 0.001), with slight changes observed thereafter (Figure 1). Similarly, MMb and MbO2 increased with concomitant decreases in DMb between 0 and 2 d, but no changes were registered thereafter (Figure 1). Lipid oxidation increased linearly during the time of air exposure (*p* < 0.001), irrespective of the proportion of pea in the concentrate (Figure 2).

The texture of the meat was not affected by the inclusion of pea either in the ST muscle or in the SM muscle (*p* > 0.05, Table 3). Regarding the chemical composition of the meat, the proportion of pea only affected the cholesterol and retinol contents (*p <* 0.05, Table 3). The meat of lambs fed 20%pea concentrate presented greater cholesterol content than the meat of lambs fed 30%pea concentrate (*p <* 0.05), but this content was similar to those observed under the rest of the treatments.

Regarding retinol, the meat of the lambs fed 10%pea concentrate presented greater retinol content than the meat of lambs fed 0%pea concentrate (*p <* 0.05), but this value was similar to those observed for the rest of the concentrates.

The proportion of pea in the concentrate had minor effects on the percentages of individual FAs in the meat (Table 4 and Table 5), affecting only the percentages of C13:0, C15:0, C17:0 and C16:1 t9 (*p <* 0.05) and the percentages of C16:0, C17:1 c9, iC18:0 and C18:2 n-6 t9, t12 (*p <* 0.10).

Regarding the sums of FAs in the meat, only the total SFAs were affected by the pea proportion (*p <* 0.01), and the concentrate with 20%pea yielded greater SFA contents than did the other concentrates (*p <* 0.05; Table 6). The proportion of pea affected the thrombogenicity index (*p <* 0.05), which was higher in the 20%pea concentrate than in the 10%pea concentrate (*p <* 0.05) and tended to affect the atherogenicity index (*p <* 0.10).

## 4. Discussion

The inclusion of pea to reduce the proportion of SBM in iso-energetic and iso-proteic concentrates involved a modification of the proportions of other ingredients, but the resulting concentrates had similar chemical compositions [16]. Numerous studies that have included pea in concentrates have used concentrates that varied by more than two ingredients [9,12,15,29], and thus, the concentrates varied in some chemical components, such as the FA profile [5]. In the present study, the FA profiles differed among the concentrates. The differences observed in the content of C16:0 are in line with the proportion of palm oil in the concentrate, which was 1.0%, 2.4%, 2.6% and 1.4% in 0%pea, 10%pea, 20%pea and 30%pea, respectively [11]. In concordance with the present results, the literature showed that when pea replaced SBM in the diets of lambs, the resulting differences in C16:0, C18:0, C18:1 c9, C18:2 n-6 and C18:3 n-3 ranged from 12% to 32%, 12% to 30%, 3% to 16%, 1% to 24% and 15% to 57%, respectively [12,15,30]. Similarly, the ingredients also contain different contents of carotenoids, especially tocopherols, causing differences in the overall contents of the concentrates. The contents in the concentrates, however, were low, and the differences in the contents of carotenoids and tocopherols among concentrates, although significant, were narrow.

The effects of pea inclusion on the colours of the RA muscle and fat deposits have seldom been evaluated in fattening lambs. The proportion of pea in the concentrate did not affect the RA colour parameters, with similar values to those previously reported in light lambs fed concentrates [17,31]. Regarding the fat colour, Lanza, Fabro, Scerra, Bella, Pagano, Brogna and Pennisi [15] found no differences when lambs were fed 40% pea or 38% fava bean compared to 18% soybean meal for 79 d [15]. Similarly, Bonanno, Tornambè, DiGrigoli, Genna, Bellina, DiMiceli and Giambalvo [3] studied the effects of four different protein sources on fat and meat colour and concluded that only fat redness and chroma were affected by the source of protein, and overall, the carcass and meat characteristics were similar to those obtained with conventional SBM. In the current experiment, the LTL muscle did not show any effect from the substitution of SBM with pea on the meat colour; this result was in line with the similar colours reported in lambs that were fed concentrates with SBM or 40%pea for 79 d [15], concentrates with 18%pea and 39%pea for 43 d [29], and concentrates with 25%pea for 49 d [30]. This lack of effect of the proportion of pea on the fat and meat colour may be due to the narrow differences in carotenoid contents among concentrates, regardless of statistical significance, in addition to the short experimental period (41 d). The time of air exposure affected all colour parameters, and haem pigments studied in the LTL, as reported in light lambs of the same breed that were fed commercial concentrates [17,32]. Usually, colour variables increase due to blooming and increased MMb contents, a plateau of approximately 5 days follows, and then discolouration occurs in the meat of light lambs [33]. Accordingly, in the current experiment, the colour variables increased at the beginning, plateaued until day 9 of air exposure when there was an increase of the Hue angle; an abrupt change is a good indicator of discolouration in the meat of light lambs regardless of the absolute value, and the greater MMb content, which indicates discolouration [34] and the end of the shelf life of meat. The lack of effects observed on either the fat or the meat colour can be considered positive because the meat colour is the main trait influencing consumer choice of light lamb [35].

The absence of any effect on the texture and shear force was in agreement with the similar values reported for lambs fed 86%pea for 48 d [3] or for lambs fed concentrates with 40%pea for 42–72 d [9,15] when compared to those fed SBM. This result indicates that the ingredients in the fattening diets of lambs have scarce effects on the texture parameters of lamb meat. The oxidative stability of meat depends on the balance between the pro-oxidant compound (i.e., total unsaturated FA, cholesterol and DMb) and antioxidant compound (tocopherols, carotenoids, … [36]) contents. In the current experiment, the mild differences observed in the unsaturated FAs and antioxidant compound contents among concentrates were not enough to elicit an effect on the lipid oxidation of the meat.

The absence of any effect on the chemical composition of the meat was expected because all diets were iso-proteic and iso-energetic; this result is in agreement with previous experiments that studied the effects of the inclusion of pea in concentrates in several doses [29,37]. The greater cholesterol content observed in the 20%pea treatment than in the 30%pea treatment agrees with the differences observed in the concentration of C16:0 in the LTL muscle (see below). This FA increases the plasma total and LDL cholesterol content [38]. However, this difference did not reach statistical significance in the plasma of these lambs at the time of slaughter [11].

The differences observed in the FA content of the concentrates were not exactly mirrored in the FA profile of the lamb meat due to the process of biohydrogenation of PUFA conducted by ruminal microorganisms [39]. The inclusion of pea had a minor effect on individual FAs in the muscle, as reported in light lambs fed pea instead of SBM in concentrates for 42–48 d [3,30]. However, when lambs were fed peas or SBM in concentrates for longer feeding periods, the most relevant FAs were affected, but these effects differed depending on the studies. The total replacement of SBM by pea for 72 d increased the C18:1 c9 and C18:3 n-3 contents but decreased the C18:1 t11 content [15], whereas the total replacement of SBM with pea for 98 d increased the C18:2 n-6 and C18:3 n-3 contents while decreasing the C16:0 and C18:0 contents [12]. The discrepancies among studies can be partially related to the amplitudes of the differences in the major FAs in the applied diets. The FAs in meat were affected only when the differences in the C16:0 and C18:0 contents in the diets of the lambs were above 30% [12] or when the differences in the C18:3 n-3 contents among diets were above 28% [12,15]. In the current experiment, the slight increase in certain individual SFAs in the meat of the lambs fed 20%pea concentrate concomitantly increased the total SFAs when compared to their counterparts, with no effects on the other FA sums. However, the total replacement of SBM in concentrate by 86%pea or 40%pea had no effect on the SFA, MUFA or PUFA contents in meat [3,15], whereas the inclusion of 24–25%pea decreased the total SFA content in meat and increased the MUFA and n-3 PUFA contents [12,30]. The slight differences in fatty acids led to differences in the thrombogenicity index, with the 20%pea concentrate yielding the greatest value, making this concentrate the least advisable. In this sense, the consumption of food with a low IA and IT has a better nutritional quality, which may reduce the risk of coronary heart disease, but no organisation has yet provided the recommended values for the IA and IT [40]. However, the impact of this difference on human health would be mild.

## 5. Conclusions

The inclusion of pea in the fattening concentrates of light lambs had no effects on fat, meat colour and lipid oxidation. Time of air exposure affected the evolution of colour and lipid oxidation, especially between 7 to 9 days. The effect on the fatty acid profile was minimal and had no effect on most FAs related to human health. However, the greater cholesterol and thrombogenicity index of the 20%pea concentrate should be considered. From the present results, the inclusion of 30%pea in concentrate would be the most advisable proportion in order to reduce the dependency on soybean meal, although the prices of the feedstuffs should be taken into account.

## Figures and Tables

**Table 1:** Fatty acid (FA) profile, carotenoids and tocopherols of the concentrates with different proportions of pea.

	0%pea	10%pea	20%pea	30%pea	s.e.	*p*-Value
n	4	4	4	4		
FA, g/100 g						
C12:0	0.08 ^c^	0.13 ^ab^	0.11 ^b^	0.15 ^a^	0.004	<0.001
C14:0	0.52 ^b^	0.68 ^ab^	0.74 ^ab^	0.77 ^a^	0.029	0.04
C16:0	34.01 ^d^	37.18 ^b^	38.15 ^a^	35.49 ^c^	0.118	<0.001
C17:0	0.19	0.15	0.17	0.21	0.014	0.52
C18:0	10.61 ^a^	8.79 ^b^	8.54 ^b^	10.61 ^a^	0.185	0.002
C18:1 c9	19.18 ^b^	23.28 ^a^	24.86 ^a^	23.19 ^a^	0.211	<0.001
C18:2 n-6	32.18 ^a^	26.95 ^b^	24.66 ^b^	26.56 ^b^	0.294	<0.001
C20:0	0.26	0.23	0.27	0.25	0.011	0.70
C18:3 n-3	2.44	2.20	2.15	2.38	0.041	0.09
C23:0	0.44	0.27	0.21	0.19	0.071	0.61
Carotenoids, µg/g DM						
Lutein	0.9 ^b^	1.2 ^b^	1.3 ^b^	1.7 ^a^	0.04	<0.001
Zeaxanthin	0.69 ^a^	0.51 ^b^	0.32 ^c^	0.47 ^bc^	0.02	<0.001
13 Z-β-carotene	0.8 ^c^	1.7 ^b^	2.2 ^a^	1.4 ^b^	0.04	<0.001
9 Z-β-carotene	0.5 ^c^	1.0 ^b^	1.4 ^a^	0.9 ^b^	0.04	<0.001
All E-β-carotene	1.1 ^b^	1.8 ^a^	2.3 ^a^	1.9 ^a^	0.06	<0.001
Tocopherols, µg/g DM						
α-tocopherol	4.4 ^a^	4.8 ^a^	4.9 ^a^	3.3 ^b^	0.07	<0.001
γ-tocopherol	10.0 ^b^	11.0 ^b^	11.4 ^b^	13.6 ^a^	0.21	<0.001
δ-tocopherol	3.4 ^c^	6.2 ^b^	7.8 ^a^	6.3 ^b^	0.06	<0.001

Within a parameter, means with different letters differ at *p* < 0.05.

**Table 2:** Effect of the proportion of pea in the concentrate on the colour and the estimator of carotenoids (SUM) of *Rectus abdominis* muscle, subcutaneous fat and perirenal fat.

	0%pea	10%pea	20%pea	30%pea	s.e.m.	*p*-Value
n	13	13	14	14		
*Rectus abdominis* muscle						
Lightness (L*)	47.8	48.0	47.3	48.1	0.3	0.81
Redness (a*)	9.1	8.5	9.0	9.0	0.2	0.71
Yellowness (b*)	9.8	10.1	9.5	10.3	0.2	0.53
Chroma (C*_ab_)	13.4	13.3	13.3	13.8	0.2	0.75
Hue angle (*h_ab_*)	47.1	49.6	45.9	49.0	1.0	0.55
Perirenal fat						
Lightness (L*)	71.4	72.3	71.6	72.4	0.3	0.66
Redness (a*)	4.8	4.2	4.6	4.3	0.2	0.56
Yellowness (b*)	11.6	11.0	11.8	11.4	0.2	0.57
Chroma (C*_ab_)	12.5	11.8	12.8	12.2	0.2	0.52
Hue angle (*h_ab_*)	67.6	69.2	68.4	69.7	0.6	0.68
SUM	120	110	123	104	5.7	0.63
Subcutaneous caudal fat						
Lightness (L*)	69.7	69.5	70.1	70.1	0.3	0.86
Redness (a*)	2.8	2.9	2.7	2.7	0.2	0.98
Yellowness (b*)	11.3	12.1	11.5	12.3	0.3	0.59
Chroma (C*_ab_)	11.6	12.5	11.9	12.7	0.3	0.62
Hue angle (*h_ab_*)	76.4	76.8	76.5	78.4	0.6	0.67
SUM	93	102	109	121	5.1	0.28

**Table 3:** Effect of the proportion of pea in the concentrate on texture, chemical composition, cholesterol, retinol and tocopherols in meat.

	0%pea	10%pea	20%pea	30%pea	s.e.m.	*p*-Value
Raw *Semitendinosus* muscle						
Shear force, N cm^−2^	41.9	43.5	42.2	41.7	0.88	0.89
Toughness, N cm^−2^	12.6	13.0	12.1	13.0	0.31	0.68
Cooked *Semimembranosus* muscle						
Stress-compression 20%, N	14.8	17.1	15.5	16.3	0.41	0.24
Stress-compression 80%, N	52.1	49.6	52.2	53.5	1.62	0.86
LTL muscle						
Dry matter, %	22.00	21.79	22.23	21.53	0.112	0.13
Crude protein, %FM	20.30	20.27	20.41	19.86	0.097	0.25
Intramuscular fat, %FM	1.65	1.67	1.93	1.72	0.051	0.26
Cholesterol, mg/g FM	0.51 ^ab^	0.51 ^ab^	0.53 ^a^	0.49 ^b^	0.005	0.02
Retinol, µg/g FM	0.023 ^b^	0.028 ^a^	0.026 ^ab^	0.024 ^ab^	0.001	0.02
α-tocopherol, µg/g FM	0.63	0.69	0.66	0.57	0.026	0.35
ɣ-tocopherol, µg/g FM	0.17	0.21	0.21	0.24	0.011	0.11

Within a parameter, means with different letters differ at *p* < 0.05.

**Table 4:** Effect of the proportion of pea in the individual saturated and monounsaturated fatty acids (FA) in LTL muscle.

	0%pea	10%pea	20%pea	30%pea	s.e.m.	Pr > F
Saturated FA, g/100 g						
C10:0	0.12	0.13	0.13	0.11	0.01	0.78
C12:0	0.24	0.20	0.28	0.24	0.02	0.37
aC13:0	0.94	1.14	0.94	1.06	0.04	0.20
C13:0	0.06 ^ab^	0.09 ^a^	0.05 ^b^	0.07 ^ab^	0.01	0.04
iC14:0	0.68	0.82	0.61	0.67	0.03	0.11
C14:0	3.38	3.12	3.46	3.05	0.09	0.32
iC15:0	0.11	0.11	0.11	0.09	0.01	0.50
aC15:0	0.66	0.68	0.63	0.67	0.02	0.83
C15:0	0.42 ^b^	0.41 ^b^	0.49 ^a^	0.47 ^ab^	0.01	0.01
DMA C16:0	0.87	0.84	0.82	0.86	0.03	0.94
iC16:0	0.13	0.11	0.12	0.12	0.00	0.36
aC16:0	0.48	0.52	0.48	0.50	0.02	0.86
C16:0	22.12	22.00	22.81	21.74	0.15	0.07
C17:0	0.99 ^b^	0.96 ^b^	1.20 ^ab^	1.27 ^a^	0.04	0.01
DMA C18:0	0.18	0.14	0.15	0.18	0.01	0.56
iC18:0	0.12	0.11	0.09	0.10	0.00	0.07
C18:0	13.29	13.40	14.12	13.78	0.17	0.28
C20:0	0.10	0.09	0.10	0.10	0.00	0.70
C22:0	0.08	0.08	0.07	0.08	0.00	0.56
C24:0	0.02	0.02	0.02	0.02	0.00	0.79
Monounsaturated FA g/100 g						
C14:1 c9	0.15	0.15	0.14	0.13	0.00	0.39
C16:1 t9	0.44 ^a^	0.41 ^ab^	0.38 ^b^	0.42 ^ab^	0.01	0.04
C16:1 c7	0.21	0.23	0.21	0.21	0.00	0.50
C16:1 c9	2.28	2.20	2.15	2.11	0.03	0.14
C17:1 c9	0.79	0.80	0.85	0.98	0.03	0.08
C18:1 t11	2.43	2.57	2.50	2.20	0.14	0.79
C18:1 c9	33.48	33.29	33.45	34.01	0.31	0.85
C18:1 t15	0.18	0.20	0.15	0.16	0.01	0.24
C18:1 c11	0.16	0.18	0.14	0.14	0.01	0.15
C18:1 c12	0.17	0.16	0.18	0.18	0.00	0.72
C18:1 c13	0.10	0.10	0.09	0.08	0.01	0.83
C18:1 t16	0.21	0.19	0.20	0.18	0.00	0.37
C18:1 c15	0.08	0.09	0.09	0.08	0.00	0.97
C24:1 c9	0.15	0.21	0.16	0.18	0.01	0.10

Within a parameter, means with different letters differ at *p* < 0.05. DMA: dimethylacetals.

**Table 5:** Effect of the proportion of pea in the individual polyunsaturated fatty acids (PUFA) in LTL muscle.

	0%pea	10%pea	20%pea	30%pea	s.e.m.	Pr > F
PUFA, g/100 g						
C18:2 n-6 t9, t12	0.17	0.15	0.15	0.13	0.00	0.06
C18:2 c9, t11	0.31	0.28	0.28	0.28	0.01	0.74
C18:2 t10, c12	0.10	0.10	0.10	0.11	0.00	0.63
C20:3 n-9	0.44	0.47	0.43	0.46	0.01	0.61
C18:2 n-6	7.84	7.72	6.89	7.38	0.18	0.25
C20:2 n-6	0.16	0.12	0.12	0.15	0.01	0.17
C20:3 n-6	0.24	0.25	0.22	0.25	0.01	0.16
C20:4 n-6	2.99	3.20	2.68	3.07	0.08	0.13
C22:4 n-6	0.28	0.29	0.25	0.28	0.01	0.32
C18:3 n-3	0.42	0.41	0.39	0.41	0.01	0.76
C20:5 n-3	0.29	0.30	0.27	0.27	0.01	0.76
C22:5 n-3	0.59	0.61	0.53	0.56	0.02	0.40
C22:6 n-3	0.26	0.28	0.24	0.27	0.01	0.71

**Table 6:** Effect of the proportion of pea in the sums and ratios of fatty acids in LTL muscle.

	0%pea	10%pea	20%pea	30%pea	s.e.m.	Pr > F
Total Saturated FA	44.98 ^b^	44.97 ^b^	46.67 ^a^	45.16 ^b^	0.20	0.01
Total Monounsaturated FA	40.19	40.15	40.11	40.47	0.33	0.98
Total Polyunsaturated FA	14.16	14.25	12.62	13.72	0.30	0.20
n-3	1.57	1.60	1.44	1.53	0.04	0.61
n-6	8.75	8.59	7.69	8.26	0.19	0.22
n-6:n-3	5.62	5.43	5.41	5.58	0.11	0.86
Atherogenicity index	0.71	0.69	0.75	0.68	0.01	0.09
Thrombogenicity index	1.32 ^ab^	1.31 ^b^	1.43 ^a^	1.33 ^ab^	0.01	0.02

Within a parameter, means with different letters differ at *p* < 0.05.

## Data Availability

The data that support the findings of this study are available from the corresponding author upon reasonable request.
